# Novel revertants of *Chlamydomonas vfl2* suggest a role for a flexible central α-helical region in centrin function in flagellar basal bodies

**DOI:** 10.17912/micropub.biology.002381

**Published:** 2026-09-16

**Authors:** Takako Kato-Minoura, Ichiro Sasaki, Ritsu Kamiya

**Affiliations:** 1 Department of Biological Sciences, School of Science and Engineering, Chuo University, 1-13-27 Kasuga, Bunkyo-ku, Tokyo, Japan

## Abstract

A *Chlamydomonas* mutant, *vfl2* , displays aberrant flagellar numbers due to an E101K mutation in the centrin gene. We isolated 21 revertants–12 intragenic and 9 extragenic–that suppress the mutant's abnormal phenotype. AlphaFold2 modeling indicates that the wild-type, mutant, and revertant proteins are structurally similar. However, the *vfl2* mutant centrin forms three salt bridges in the central region of the core α-helix, whereas the wild type and all intragenic revertants have only two salt bridges or disrupted helix continuity. These results indicate that flexibility of the central helix, maintained by limited electrostatic interactions, is important for proper centrin function.

**Figure 1. Summary of vfl2 revertant classes and associated mutations:** A, Genotypes of the *vfl2* revertants. B, wild-type overall centrin (Vfl2) structure (PDB: 3QRX, Sosa et al., 2011); C, magnified mid portion of wild-type centrin (boxed in B); D–J, AlphaFold2-predicted centrin structures in *vfl2* (D), *vfl2-R201* (E), *vfl2-R206* (F), *vfl2-R214/R221* (G), *vfl2* - *R220/R222* (H), *vfl2-R202/R215/R217* (I), *vfl2-R205* (J). In B, orange spheres indicate Ca ^2+^ ions. Light green, dark green, light teal, and deep teal indicate the 1st–4th EF-hand motifs, respectively. In the central α-helices in B–J, acidic/basic amino acids are shown as a stick model, with acidic amino acids (E or D) colored red and basic amino acids (K or R) colored blue. Other mutated amino acids are colored orange. In C–J, salt-bridge distances are shown in Å, and the specific amino acid variations are shown at the bottom of each panel. The numbers in the schematic diagram of panel C indicate the amino acid positions in the wild-type primary sequence, illustrating their spatial arrangement along the pitches of the α-helix. Note that the amino acid at position 98 is not shown except in panel I, as this residue lies on the back side of the helix and is not visible in the ribbon diagrams. K: alignments of the amino acid sequences of the mid portion of the central α-helix in wild type, *vfl2* , and type 2 and type 3 revertants.

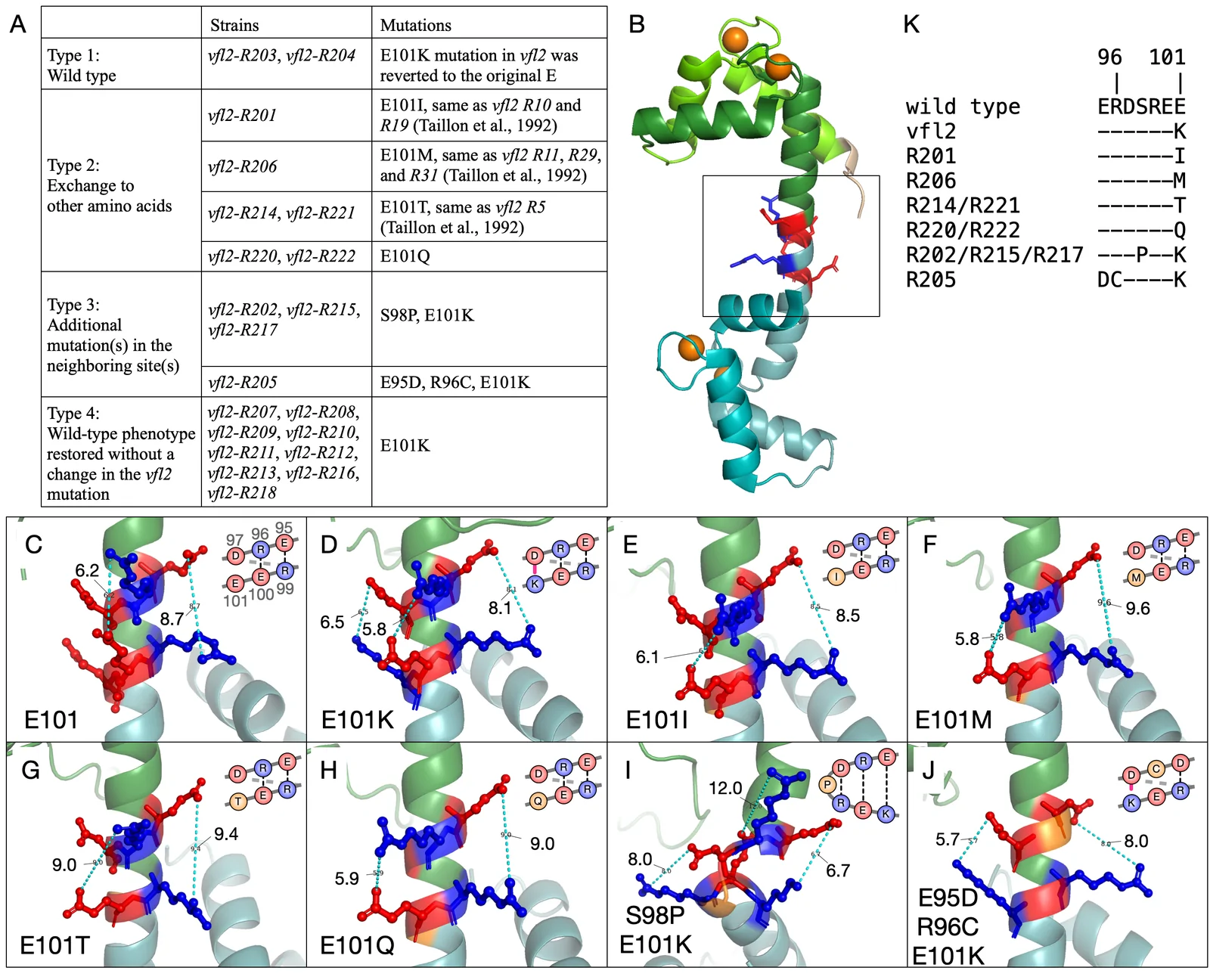



## Description

Centrin is a highly conserved eukaryotic protein of ~ 20 kDa with four Ca ^2+^ -binding EF-hand motifs. In animal cells, centrin localizes to the centriole, the core structure of the centrosome, and plays essential roles in mitotic spindle formation and cytoplasmic microtubule organization. In ciliated cells, the centriole becomes a basal body and functions as the template for axonemal assembly. Centrin is thus essential for both cell division and ciliation (flagellation).

In the unicellular biflagellate green alga *Chlamydomonas* , centrin is expressed from the gene *vfl2* . It mainly localizes to three distinct sites in interphase cells: fibers connecting the nuclei and basal bodies, distal striated fibers (DSF) that connect the two basal bodies, and the transition zone located between the basal body and the flagellum (Wright et al., 1985; Schulze et al., 1987; Salisbury et al., 1988; Sanders and Salisbury, 1989). In most cases, centrin is contained in fibrous structures, which can contract at higher Ca ^2+^ concentrations (Wingfield and Lechtreck, 2018). Centrin in the transition zone has been suggested to participate in flagellar shedding (Sanders and Salisbury, 1989), and the DSF centrin to function in changing the angle between the two flagella in a Ca ^2+^ -dependent manner (Salisbury et al., 1987; Hayashi et al., 1998).

The *Chlamydomonas* mutant *vfl2* , which displays aberrant flagellar numbers, carries a centrin-gene mutation causing a Glu-to-Lys substitution at position 101 (Kuchka and Jarvik, 1982; Taillon et al., 1992). Taillon et al. (1992) mutagenized this mutant and obtained 19 revertants that apparently recovered wild-type flagellation. Ten of these had changes at Lys101 to one of five amino acids (Taillon et al., 1992). The present study extended previous work by isolating 21 additional phenotypic revertants and identifying several novel amino acid changes ( Figure 1A ). All revertants displayed near-normal swimming phenotype due to recovery of normal flagellar number. Sequence analysis revealed four classes: type 1, in which the wild-type sequence was restored; type 2, in which Lys101 was replaced by an amino acid other than the original glutamic acid; type 3, which harbored additional mutation(s) while retaining the original E101K mutation; and type 4, in which no mutation was found other than E101K. Types 1 through 3 are intragenic revertants, whereas type 4 likely reflects extragenic suppression. Some classes match those previously reported, whereas others represent newly identified revertant types (see also Extended Data Figure 1).

We focus here on type 2 and type 3 mutants with intragenic changes, although type 4 mutants are also interesting and warrant future studies, as these revertants indicate mutations in non-centrin proteins. As shown in Figure 1E —J, all amino acid changes in type 2 and type 3 occurred in the central portion of the central α-helix in centrin. Within this region, electrostatic interactions occur between different amino acid side chains. For example, in the two pitches of the α-helix N-terminal to position E101, attractive interactions occur between E95 and R99, and between R96 and E100, while a repulsive interaction occurs between D97 and E101 ( Figure 1B, C). In contrast, in *vfl2* centrin, there are three attractively interacting amino-acid pairs: E95-R99, R96-E100, and D97-K101 ( Figure 1D ). This suggests that the α-helix around position 101 is more stabilized in *vfl2* than in wild type, although AlphaFold2 (Jumper et al., 2021) predicts similar structures for wild-type and *vfl2* centrins. All type 2 and type 3 revertants isolated in this study have either only two attractive amino-acid pairs or a proline kink in this region ( Figure 1E —J). In other words, no revertants were isolated that retained the three pairs of oppositely charged amino acids present in *vfl2* . These results suggest that appropriate centrin flexibility—arising from limited electrostatic interaction or disruption of helix continuity—is important for centrin function at the flagellar base.

Notably, among the four types of amino acid substitutions at position 101 reported by Taillon et al. (1992)—specifically I, M, T, and N—our study isolated all but E101N. Although we did not isolate this mutation in our screen, an E101N substitution does not introduce a basic side chain and therefore cannot form the third salt bridge with D97, leaving the central helix with only two salt bridges. Thus, all revertants obtained by Taillon et al. (1992) likely have centrins with a more flexible central helix than the original *vfl2* centrin, consistent with our proposed model.

Taillon et al. (1992) reported structural deficiencies in the DSF in some of their revertants. Although we have not examined the DSF structures in our isolated revertants, they may harbor similar defects. How central-helix flexibility specifically affects centrin function in regulating basal body number and DSF structure remains an important subject for future studies.

Based on sequence analyses of revertants, we propose that excessive stabilization of the centrin α-helix around position 101 is detrimental to its function, and that some flexibility in this region may be necessary. This idea contrasts with the proposal of Taillon et al. (1992) that E101 stabilizes the centrin structure by forming a salt bridge with another basic amino acid and that this stabilization is important for centrin function. In calmodulin (CaM), a homolog of centrin, the middle region of the corresponding central helix functions as a "flexible tether" crucial to its function (Persechini et al., 1988a). In CaM, the central region is almost entirely composed of negatively charged amino acids and therefore barely forms an α-helix (Persechini et al., 1988b; Barbato et al., 1992, for review, see Nelson and Chazin, 1998). Thus, some flexibility in the central region of the central helix may also be essential for centrin function. Having only two salt bridges in centrin's central helix may therefore be important for allowing this flexibility; this could be tested by generating mutants with additional salt bridges near position 101 in future studies. 

## Methods

**Revertant isolation**

The mutant *vfl2* (Kuchka and Jarvik, 1982) was cultured in liquid TAP medium (Gorman and Levine, 1965) for 3 days, placed in a Petri dish, irradiated with a UV lamp (20 W) from 40 cm above for 2 min, and transferred to test tubes with 8 mL liquid TAP. After being kept in the dark for 1 day to prevent photo-recovery, the upper 0.5 mL of each test tube, containing swimming cells, was transferred to another test tube with 8 mL of fresh medium. This process was repeated a few times. Finally, the cells swimming on the upper surface of each test-tube culture were inoculated onto TAP agar plates to produce single colonies. Each clone was cultured in a multi-well plate with TAP medium for motility assessment under an inverted microscope. Strains that showed near-wild-type motility were saved as revertants.

**Genomic DNA sequencing**

Genomic DNA from *vfl2* and its revertants was purified using the method of Rochaix et al. (1988), with some modifications. The coding region was then amplified using primers 5vfl2-F and 3vfl2-R (see Reagents). PCR products were purified using Wizard® SV Gel and PCR Clean-Up System (Promega), and their sequences were determined using primers 5vfl2-F and 5vfl2-F2 (see Reagents). Sequencing was entrusted to Macrogen Japan.

**3D structure prediction**

AlphaFold2 (Jumper et al., 2021) was used to predict the 3D structure of *vfl2* and revertants centrins, then plotted in PyMOL (DeLano Scientific,https://www.pymol.org).

## Reagents

**Table:** 

PRIMER NAME	SEQUENCE
5vfl2-F	5'-AGTCTGCTGCTGGAGCTCAGACC-3'
3vfl2-R	5'-AATTTATTATGTGCCGTGCTGTGCAC-3'
5vfl2-F2	5'-TGTGGGCATAGCGGTGACAAA-3'

**Table:** 

STRAIN	GENOTYPE	AVAIRABLE FROM
CC-125	Wild type	*Chlamydomonas* Resource Center
*vfl2*	E101K in Vfl2	*Chlamydomonas* Resource Center (Kuchka and Jarvik, 1982; Taillon et al., 1992)
*vfl2-R201~R222*	see Figure 1A	All revertants isolated in this study will be deposited in the *Chlamydomonas* Resource Center.

## Data Availability

Description: Sanger sequencing data of vfl2 and 21 revertants. Only partial regions (bases 286 to 306), where the A at the start codon is 1, are shown. The red arrows indicate the vfl2-type SNP that results in the E101K mutation. The blue arrows indicate additional changes, grouped by revertant type.. Resource Type: Image. DOI: https://doi.org/10.22002/qpeqx-0qg84
